# Application of High-Performance Liquid Chromatography Combined with Fluorescence Detector and Dispersive Liquid–Liquid Microextraction to Quantification of Selected Bisphenols in Human Amniotic Fluid Samples

**DOI:** 10.3390/ijerph20010297

**Published:** 2022-12-24

**Authors:** Szymon Szubartowski, Tomasz Tuzimski

**Affiliations:** 1 Department of Physical Chemistry, Medical University of Lublin, Chodźki 4a, 20-093 Lublin, Poland; 2 Doctoral School of Medical University of Lublin, Medical University of Lublin, Chodźki 7, 20-093 Lublin, Poland

**Keywords:** bisphenol A (BPA), bisphenol A analogues, bisphenol A diglicydyl ether (BADGE), high-performance liquid chromatography (HPLC), fluorescence detector (FLD), dispersive liquid–liquid microextraction (DLLME), amniotic fluid

## Abstract

Bisphenol A (BPA) is a widely produced chemical worldwide found in numerous everyday products. Its endocrine-disrupting properties and omnipresence have aroused concern and led to several restrictions on its use. These restrictions and growing public awareness about the toxicity of BPA have resulted in market products labeled ”BPA-free”, with BPAs often being replaced by other bisphenols. This is why constant biomonitoring of bisphenol levels in various body fluids and tissues is essential. In this study, we propose the use of simple, cost-effective high-performance liquid chromatography coupled with the fluorescence detector (HPLC-FLD) method for the determination of simultaneously selected bisphenols in amniotic fluid. For the sample preparation, a fast, simple, and ”green” dispersive liquid–liquid microextraction (DLLME) method was used, achieving mean recovery values in the range of 80.9–115.9% with relative standard deviations below 12% for all analytes. Limits of quantification (LOQs) determined in the amniotic fluid matrix ranged from 6.17 to 22.72 ng/mL and were obtained from a calibration curve constructed using least-squares linear regression analysis for all cases. The presented sample preparation procedure can be easily adopted for LC-MS analysis.

## 1. Introduction

From the 1950s to 2015, plastic waste production amounted to 6.3 billion tons, and it is predicted to reach 26 billion tons. Bisphenols are used as additives in the production of plastic to obtain the desired properties in products [1]. Bisphenol A (BPA) is one of the monomers produced in the highest volumes worldwide, and it is used in the production of polycarbonate plastics, epoxy resins for the lining of metal cans, and numerous products for everyday use [2]. Results of experiments have shown that BPA can migrate to food, and migration increases at higher temperatures and lower pH [3]. BPA can be detected in various human tissues and body fluids, such as urine, blood (including umbilical cord blood), breast milk, follicular fluid, amniotic fluid, sweat, and solid tissues, such as adipose tissue, the brain, and liver [2,3]. BPA is an endocrine disruptor, which means that it disturbs the proper function of the endocrine system by acting as the endogenous hormone 17-β-estradiol. Despite being an agonist 1000 to 10,000 times weaker than human hormones, BPA exhibits an adverse effect on hormonal balance. In addition to endocrine disruption, BPA is linked to obesity, type II diabetes, heart diseases, hepatotoxicity, neurotoxicity, immunotoxicity, and mutagenicity [3]. The omnipresence and toxicity of BPA have attracted the attention of food safety control agencies around the world, such as the European Food Safety Authority, which imposed restrictions on its use and banned it in infants’ products, such as feeding bottles [4]. The specific migration limit (SML), which is defined as the maximum amount of a given substance permitted to be released from a material or article into food or food simulants, has been set at 0.05 mg/kg for BPA in food, and it should not migrate from products intended for infants and young children [5]. Restrictions on the use of BPA have led to alternatives that are often BPA analogues; e.g., bisphenol S (BPS), bisphenol (BPF), and bisphenol AF (BPAF) have entered the market. However, BPA analogues exhibit similar toxic properties, such as endocrine-disrupting properties, reproductive toxicity, neurotoxicity, and cytotoxicity [6,7,8]. Currently, only a few bisphenol A replacements are under restrictions. The SML for BPS was set at 0.05 mg per kilogram of food [4], and the sum of bisphenol A diglicydyl ether (BADGE), BADGE∙H_2_O, and BADGE∙2H_2_O cannot exceed 9 mg/kg in food or food simulants or 9 mg/6 dm^2^ for items that are containers, comparable to containers, or that can be filled to a capacity of less than 500 mL or more than 10 L and for sheets, films, or other materials that cannot be filled and for which it is impracticable to estimate the relationship between the surface area and the quantity of foodstuffs in contact. Similarly, the SMLs for the sum of BADGE∙HCl, BADGE∙H_2_O∙HCl, and BADGE∙2HCl in the two contexts described above have been set at 1 mg/kg and 1 mg/6 dm^2^, respectively [9].

The omnipresence of bisphenols in everyday life has necessitated the development of methods for their biomonitoring in various matrices, both in body fluid and solid tissues, such as urine, serum, breast milk, sperm, and hair. This has led to the development of numerous sample preparation techniques, such as the Quick Easy Cheap Effective Rugged Safe (QuEChERS) procedure [10,11,12,13,14], liquid–liquid extraction (LLE) [15,16,17], solid-phase extraction (SPE) [18,19,20,21,22,23,24], on-line SPE [25,26], dispersive liquid–liquid microextraction (DLLME) [27,28,29], and more. The low concentration of analytes found in biological samples combined with complex matrices requires not only effective sample preparation but also a very sensitive and reliable separation technique, such as liquid chromatography or gas chromatography. The most common technique is liquid chromatography coupled with tandem mass spectrometry (LC-MS/MS), with which high sensitivity and reliability can be achieved for the obtained results [30].

An interesting sample preparation technique that could be widely used in the biomonitoring of bisphenols is dispersive liquid–liquid microextraction (DLLME). DLLME was first presented in 2006 by Assadi and co-workers for the isolation of polyaromatic hydrocarbons (PAHs) from water samples [31,32]. In the DLLME procedure, the extraction mixture consists of a dispersive solvent, which must be miscible with water, and an extraction solvent, which must have a higher density and be immiscible with water, such as chloroform, dichloromethane, or 1,2-dichloroethane. Compared to other popular techniques, such as the QuEChERS procedure or SPE, DLLME is more time-effective, does not require sophisticated laboratory equipment, and only consumes a small amount of harmful solvents. This is why DLLME is classified as a “green” method. DLLME was successfully applied in bisphenol analysis in biological samples, which is described in detail in Section 4. In our previous study [33], we proposed an SPE-based method using Oasis HLB columns (400 mg) for 11 bisphenols and analyzed them with high-performance liquid chromatography coupled with a fluorescence detector (HPLC-FLD). In this study, we propose a DLLME-based method for the simultaneous determination of selected bisphenols using HPLC-FLD. The selected analytes have been previously found in other biological matrices, such as urine or breast milk [30]. Compared to our previous study, we obtained similar or higher recovery values and limits of quantification (LOQs) while keeping the same small sample volume, which is crucial considering the low availability of amniotic fluid. Furthermore, the sample preparation time and solvent consumption were significantly reduced. HPLC-FLD might be a cheaper alternative for bisphenol analysis of biological and environmental samples thanks to its high sensitivity to signal amplification and high selectivity [23,24].

## 2. Materials and Methods

### 2.1. Bisphenol Standards

All five bisphenol standards—4,4-Methylenediphenol (bisphenol F (BPF)), 1,1-Bis(4-hydroxyphenyl)ethane (bisphenol E (BPE)), 2,2-Bis(4-hydroxyphenyl)hexafluoropropane (bisphenol AF (BPAF)), 1-Chloro-3-[4-[2-[4-(3-chloro-2-hydroxypropoxy)-phenyl]propan-2-yl]phenoxy]propan-2-ol (BADGE∙2HCl), and 4,4-(1,4-Phenylenediisopropylidene) (bisphenol P (BPP))—were obtained from Sigma-Aldrich (Bellefonte, PA, USA) and their purity was ≥98%. All studied bisphenols, along with their structures, IUPAC names, log *p* values, and pKa values, are presented in Table 1.

Bisphenol standards were diluted in methanol and stored in the freezer at −23 °C. The proper mixture for spiking was prepared by combining all bisphenol solutions immediately before the DLLME procedure. Proper concentrations were achieved by dissolving solutions in methanol and used both for recovery studies and for calculating the limits of detection and quantification (LODs and LOQs).

### 2.2. Chemicals Used during the Instrumental Analysis and DLLME Procedure and Laboratory Equipment

Methanol (MeOH) and acetonitrile (ACN) with LC-MS-grade purity were obtained from E. Merck (Darmastadt, Germany). LC-MS-grade water, acetone, chloroform (CHCl_3_), dichloromethane (CH_2_Cl_2_) with purity for HPLC ≥ 99.8%, and formic acid (HCOOH) with purity ≥ 98% were obtained from Sigma-Aldrich (St. Louis, MO, USA). Deionized water was produced on a continuous basis using a Hydrolab System (Gdańsk, Poland).

Disposable, “latex-free” 5 mL syringes and needles were obtained from a local pharmacy. To avoid contamination, especially from the plastic syringes, optimized procedures were performed for LC-MS-grade water samples and no contamination was found. Syringes were calibrated with an Eppendorf automatic pipette (100–1000 µL).

### 2.3. Instrumental Analysis

Chromatographic analysis was performed on an Agilent 1200 system consisting of a quaternary pump, an autosampler with a thermostat (1260 Infinity II Vialsampler), a thermostated column compartment, and a fluorescence detector (Agilent Technologies 1260, FLD, Wilmington, DE, USA). The samples were thermostated at 4 °C.

The chromatographic conditions were slightly changed based on previously published work [33]. The separation was conducted on a Scherzo SM-C18 (150 mm × 4.6 mm) column with a 3 µm particle size (Agilent Technologies, Wilmington, DE, USA), thermostated at 22 °C. The mobile phase consisted of 50 mM formic acid (HCOOH) in water (component A) and 50 mM HCOOH in ACN (component B). The gradient elution was as follows: 0–15 min from 40 to 75% component B; 15–15.5 min from 75 to 85% component B; 15.5–21 min isocratic elution 85% B. The flow rate was 0.45 mL/min.

After every sample injection, the column was washed with 100% component B with a 1.0 mL/min flow rate for 10 min, and then the column was conditioned with the isocratic elution with the initial composition (40% B) for 15 min.

### 2.4. Method Validation

The validation study was performed using spiked human amniotic fluid samples and included evaluation of the selectivity extraction recovery precision and accuracy, linearity, and limits of detection (LODs) and quantification (LOQs).

### 2.5. Selectivity

The selectivity was evaluated by comparing the chromatograms from the mixture of bisphenol standards, blank amniotic fluid (AF) samples from different sources (averaged amniotic fluid sample obtained by mixing 10 AF samples from different women), and the spiked averaged AF sample to investigate the potential interference with the signals of the analytes. The identification was performed on an FLD detector at four different wavelengths simultaneously and the optimal one was chosen. The identification of bisphenols was based on the retention times of the analytes. HPLC analyses were repeated three times.

### 2.6. Linearity

**Figure 1:**
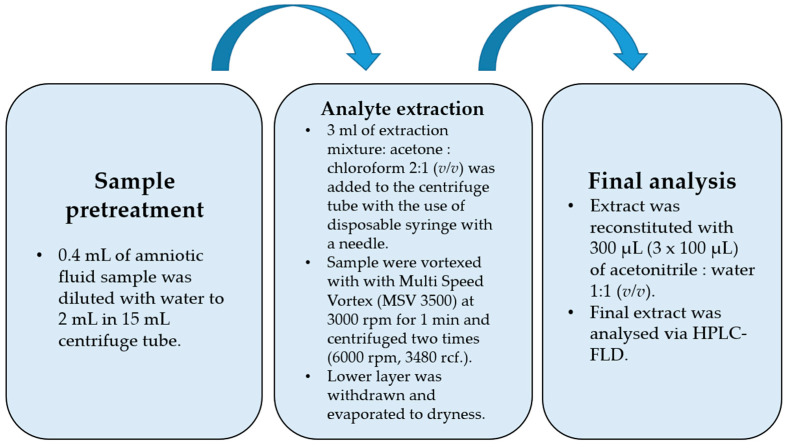
Flowchart of final optimized DLLME procedure.

**Figure 2:**
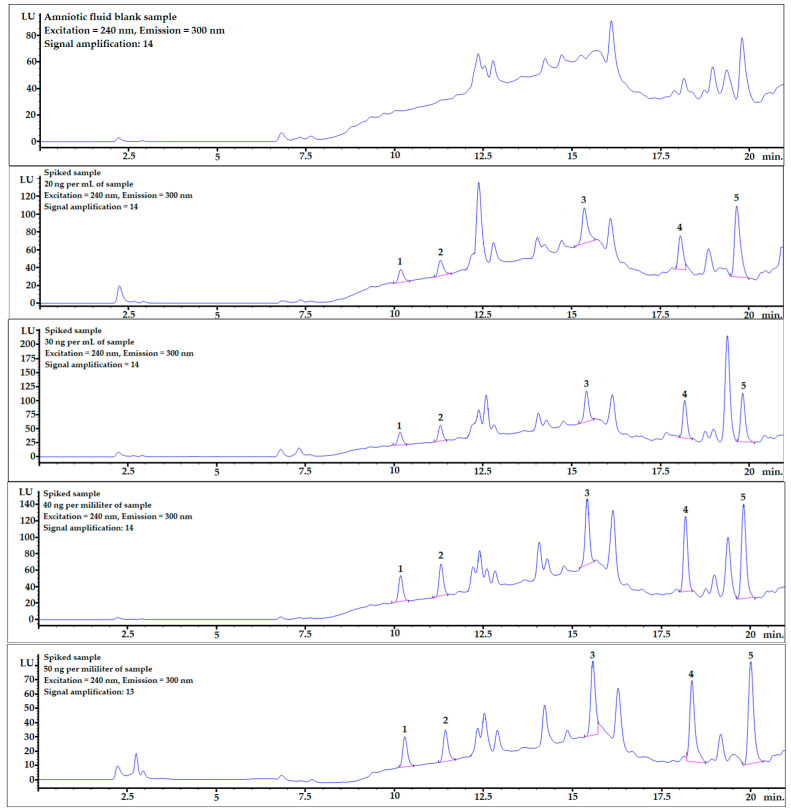
Examples of chromatograms obtained during recovery studies for each spiking level: 0 (averaged blank sample), 20, 30, 40, and 50 ng per mL of sample (from top to bottom). 1—BPF, 2—BPE, 3—BPAF, 4—BADGE∙2HCl, 5—BPP.

**Figure 3:**
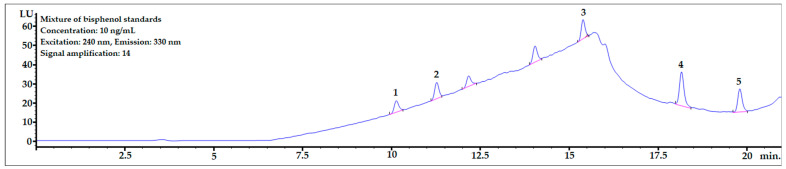
The chromatogram of the mixture of bisphenol standards at 10 ng/mL dissolved in MeOH. 1—BPF, 2—BPE, 3—BPAF, 4—BADGE∙2HCl, 5—BPP.

The calibration curves were constructed in averaged blank-matrix AF samples at six concentration levels ranging from 1 to 40 ng/mL. For further calculations, the lowest concentration (1 ng/mL) was rejected and the final concentration range used for the calculation of LOD and LOD values was 5–40 ng/mL. Several final averaged blank-matrix AF samples (after the DLLME procedure, please see Figure 1) were divided into equal parts and provided with the same amounts of the bisphenol standard mixture to prevent adulteration due to unequal dilution and to properly homogenize the sample. The mixture of standards was 5% of the final volume. Calibration curves were studied by correlating the peak area to the concentration. To evaluate linearity, coefficients of determination (R^2^) were analyzed. It was originally planned to also quantify BPA and BPE; however, due to their poor separation from the matrix components, they were rejected. As shown in Figures 2, 3, and 5, their peaks were found to remain at ~12 min and ~14 min in the analysis.

The LOD and LOQ values were obtained using the equations 3.3× (SD/S) and 10× (SD/S), respectively, where SD is the standard deviation of the response (peak area), and S is the slope of the calibration curve. Analytes were identified on the basis of time retention. The signal amplification was set at 14. HPLC analysis was repeated three times.

### 2.7. Extraction Recovery Studies

Mean recoveries were evaluated at four different concentration levels: 20, 30, 40, and 50 ng/mL of the sample. Mean recovery values were obtained from six replicates (*n* = 6) for every spiking level and calculated with the formula:

$$\mathrm{Recovery}\;(\%)=\frac{A}{B}\times 100\%.$$

*A.* Peak area of the analyte obtained after procedure where sample was spiked before DLLME extraction;

*B.* Peak area of analyte obtained after procedure where sample was spiked after DLLME extraction directly into vial.

Relative standard deviation was calculated as follows:

$$\mathrm{RSD}\%=\frac{C}{D}\times 100\%.$$

*C.* Standard deviation of the recovery (%);

*D.* Mean recovery (%).

### 2.8. Optimized DLLME Procedure for Amniotic Fluid Samples

Amniotic fluid (0.4 mL) was transferred into a 15 mL falcon tube and diluted to 2 mL with deionized water. For the spiked samples, a 20 µL mixture of bisphenol standards was added before dilution (20 μL of MeOH for blank samples). Then, 3 mL of extracting mixture (acetone: CHCl_3_ 2:1 (*v*/*v*)) was withdrawn with a disposable syringe fitted with a needle, injected into the falcon tube with a 2 mL water sample, and vortexed with a Multi Speed Vortex (MSV 3500, Bionovo, Poland) at 3000 rpm for 1 min. The tube was then centrifuged two times (6000 rpm, 3480 rcf). The lower layer was collected with an automatic pipette, evaporated to dryness, and then reconstituted in 300 µL of ACN/H_2_O (1:1, *v*/*v*).

### 2.9. Human Amniotic Fluid Sample Collection and Storage

The samples of amniotic fluid (AF) were obtained during amniocentesis between July 2021 and September 2021 from the Department of Obstetrics and Pathology of Pregnancy, Medical University of Lublin, Poland. A total of 23 mL of AF was collected from women in the 15th–26th weeks of pregnancy, with 3 mL being donated for this study and 20 mL used for genetic testing. Samples were collected in bisphenol-free tubes and frozen at −23 °C until analysis.

This study was approved by the Bioethics Committee at the Medical University of Lublin, Poland (Resolution of the Bioethics Committee at the Medical University of Lublin No. KE-0254/239/2021).

## 3. Results

### 3.1. Optimizing the Procedure

The flowchart of the final DLLME procedure is shown in Figure 1. While optimizing the procedure, the extraction mixture volume and composition were checked. Acetone was used as a dispersive solvent due to its properties, which include miscibility with water and organic extracting solvents, and combined with dichloromethane and chloroform. Based on previously published papers [27,28,29], acetone was chosen as the extracting solvent. The preliminary recovery studies were performed for the spiked samples with 50 ng per mL of sample (*n* = 6), and the results are shown in Table 2. The best recovery values combined with the most satisfactory chromatograms were obtained using a mixture of acetone and chloroform. For this optimal variant of DLLME, additional modifications were performed in the following experiments. Lowering the pH value to 5 did not change recoveries positively due to the pKa levels of the examined bisphenols, which indicated that the bisphenols had to be in fully non-ionized forms at a nearly neutral pH value (from 7 to 8), apart from BPF (see Table 1). Salting out did not have a significant effect on recovery values. The Multi Vortex System provided a thorough and repeatable mix of the two phases.

The procedure was begun with a fivefold dilution of 0.4 mL of the amniotic fluid sample (AF) with water to reach 2 mL. Then, the extraction process was performed with the disposable syringes equipped with disposable needles. After vortex mixing, when the analytes were extracted into the chloroform phase and centrifuged, the lower chloroform phase (higher density than water) was withdrawn, evaporated to dryness, and then reconstituted in a 300 µL (3 × 100 µL) mixture of ACN:H_2_O (1:1, *v*/*v*).

### 3.2. Recovery Studies

Mean recovery levels (%) and relative standard deviations (RSD%) were studied at four different spiking levels: 20, 30, 40, and 50 ng per mL of sample (*n* = 6). The chromatograms, including one example of each spiking level and mixture of bisphenol standards (10 ng/mL), are shown in Figure 2.

The mean recovery values and RSD% are presented in Table 3.

The recovery values ranged from 80 to 120%. Recovery levels above 100% might have been caused by the matrix effect, which can be evaluated by analyzing linear regression (Table 4). All relative standard deviations were set below 12%, which confirmed the repeatability and reliability of the optimized procedure.

Example chromatograms with the application of gradient elution for the averaged blank sample and for each spiking level are shown in Figure 2.

### 3.3. Chromatographic Conditions

The chromatographic method was based on one previously published [33]. Separation was performed on a Scherzo SM-C18 column and, thanks to the applied gradient, all analytes were eluted in 21 min. Detailed gradient settings are described in the Section 2.

The chromatogram of the mixture of bisphenol standards dissolved in methanol obtained in the analysis is shown in Figure 3. All bisphenols were well-separated from each other, and peak shapes were symmetric (symmetry factor in the range of 0.85–1.1).

### 3.4. Detection Conditions

The fluorescence detector used as part of the detection technique provided crucial advantages in the biomonitoring of the bisphenols, such as high sensitivity thanks to signal amplification from 1–18 and high specificity: only compounds containing fluorophores in their structures were detected, which might be helpful in complex matrices. The FLD enabled analysis across four different optimal wavelengths simultaneously, making it possible to obtain four different chromatograms and choose the most optimal one.

Some bisphenols are described as a fluorescent molecules [34]. The bisphenols exhibited different fluorescent properties. The detector response under the studied conditions was much stronger for BADGE∙2HCl, BPP, and BPAF than for BPF and BPE. The better detection might have resulted from differences in structures, such as, comparing their structures to BPA, an additional phenol ring in BPP; six fluorine atoms replacing six hydrogen atoms in two CH_3_ groups connected with a carbon atom linking two phenyls in BPAF; and two 1-chloro-2-hydroxypropyl substituents attached to the phenolic groups for BADGE∙2HCl. The detector responses for BPF and BPE were similar, and their structures differed only in the numbers of -CH_3_ substituents attached to the carbon atom connecting the two phenyl rings. The FLD detector settings, such as excitation wavelength and emission wavelength, were selected based on a study of the fluorescence properties of BPA, BPS, and BPF [34]. Then, thanks to the possibility of measuring at four different excitation wavelengths with the FLD, four excitation wavelengths were selected (225, 230, 235, and 240 nm), while the emission wavelength was set to 300 nm during all analyses. The excitation wavelength of 240 nm was selected as the optimal one after evaluating the peak areas and the quality of the obtained chromatograms. 

However, identification and quantification were achieved by comparing time retention only, and signal amplification might have increased not only the analytes’ peaks but also the components of the matrix, which might have led to overestimation of the results due to the co-elution of the matrix interferences. This can be overcome by constructing calibration curves for the averaged blank sample or the reference materials when the matrix effect can be estimated. Details of the detector settings can be found in the Section 2.

**Figure 4:**
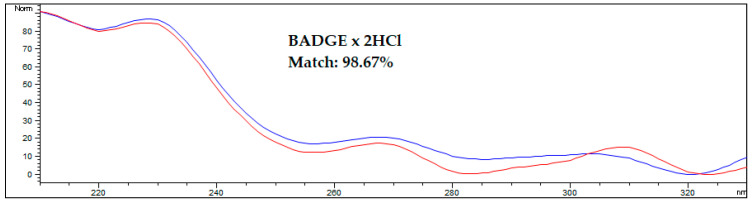
Comparing the spectrum of the BADGE∙2HCl standard with the spectrum acquired during HPLC-FLD analysis of spiked amniotic fluid samples after optimized DLLME procedure. The blue line represents the standard solution in methanol, the red line represents the spiked human amniotic fluid sample. Results were obtained for a sample spiked at 50 ng/mL.

An interesting approach for analytes with bands that overlap with matrix components is to confirm the proofs of their identity by comparing the spectra obtained from the analyte standard solution and from the analyte found in the spiked or real sample. A comparison of spectra from the standard and the analyte in the spiked AF sample is shown on Figure 4.

### 3.5. Optimization of the HPLC-FLD Procedure for Quantitative Analysis

The calibration curves constructed from the concentration ranged from 5 to 40 ng/mL and exhibited satisfactory coefficients of determination above 0.9816; for four of five analytes, the R^2^ value was above 0.99.

BPF and BPE analytes were well-separated from the matrix components (see Figure 2), and the matrix effect was very low (see linear regression column in Table 4). The LOQ values were also at satisfactory levels for the quantification of these analytes in real AF samples.

The LOQ value of BPAF may have been too high to quantify, but the LOD level might be sufficient for qualitative determination of this bisphenol in real AF samples. When comparing the structures of individual bisphenols, the detection of BPAF should indicate that it has one of the largest structures. However, when this analyte was quantified in amniotic fluid samples, the BPAF signal was suppressed. Therefore, the LOD and LOQ values of this bisphenol were the highest among the analyzed compounds.

In contrast to what was described above, the matrix effects for the last two analytes, BADGE∙2HCl and BPP, were much stronger, which is clearly noticeable in Table 4 and Figure 2. However, the detector response was sufficient to obtain very satisfactory LOD and LOQ levels. Moreover, the matrix effect can be omitted by changing the chromatographic conditions and fully separating the analyte from matrix interferences or submitting the procedure to LC-MS analysis.

### 3.6. Applying the Procedure for Sample Spiked with Amount Close to Real Concentration Found in Human Body Fluids

**Figure 5:**
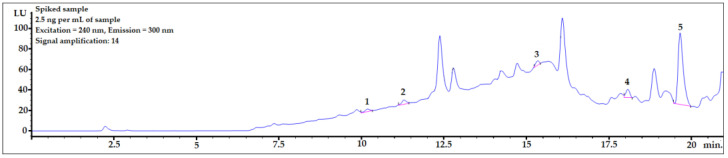
Amniotic fluid sample spiked with 2.5 ng per mL of sample. 1—BPF, 2—BPE, 3—BPAF, 4—BADGE∙2HCl, 5—BPP.

To check whether the procedure would be useful for biomonitoring bisphenols in real human AF samples, the presented DLLME procedure with HPLC-FLD analysis was performed for a sample spiked at 2.5 ng per mL of sample, which is the concentration level observed in human samples. As shown in Figure 5, the peaks of all the analytes were clearly visible. The LOD value of BPP was very close to the final analyte concentration in the sample. Therefore, to identify a BPP analyte with lower concentrations in amniotic fluid samples, it would be necessary to set a higher amplification for the analyte signals using an FLD detector. However, because of the strong matrix effect (see linear regression values for BPP in Table 4), the response from the detector might be too strong for reliable analysis. BPF, BPE, and BADGE∙2HCl could be detected (see LOD and LOQ values in Table 4) using the presented method at this concentration level.

Most analyses presented in this work were performed using signal amplification set at 14. To determine lower concentrations, stronger signal amplification (up to 18) can be applied. For analytes with low matrix influence, stronger signal amplification may lower the limits of quantification to under 1.0 ng/mL when using the calculation method described in the Section 2.6.

## 4. Discussion

A comparison of the presented DLLME technique for HPLC analysis and examples of previously published methods using DLLME is shown in Table 5. Unlike the technique from this work, all other DLLME procedures presented in Table 5 included an enzymatic hydrolysis stage with β-glucuronide to determine the total level of bisphenols. Bisphenols administered orally were mostly conjugated with glucuronic acid and extracted with urine after 6 h [35]. However, owing to less effective conjugation in fetuses and newborns, biomonitoring of free forms in AF samples is important. DLLME extraction with HPLC-FLD analysis offers a quick, solvent-saving, ”green” method for determination of a group of bisphenols. This method does not require a derivatization step, unlike GC-MS. LOD and LOQ levels differed significantly, which was caused by the different calculation method. The other advantage of the presented procedure is the very low sample volume compared to urine studies, which is crucial for samples with very low availability and invasive sampling [27,28].

Compared to our previous study [33]:

- The sample volume stayed the same (0.4 mL);
- The method was optimized for 5 bisphenols, while the previous study utilized a method optimized for 11 bisphenols. However, in the previous study, some bisphenols were not fully separated from each other (for example, BPAP and BPAF). For recovery studies, mixtures of 11 bisphenols were divided in two parts;
- The SPE procedure was more time- and solvent-consuming;
- Recoveries and repeatability remained at acceptable levels;
- The LOQ values were 3.2–12.6 ng/mL for [33] and 6.17–22.72 ng/mL for this work.

To sum up, the relatively low complexity of the matrix and the high recovery values with acceptable repeatability indicate that this procedure is suitable for determining bisphenols in real amniotic fluid samples. Increasing the signal amplification should even provide LOQ levels below 1.0 ng/mL, especially for analytes 1, 2, and 3, which were fully separated from matrix components. Applying this procedure with other instrumental techniques, such as LC-MS equipped with a triple quadrupole, or changing the stationary might represent solutions for separation problems with BADGE∙2HCl and BPP. To biomonitor total bisphenol concentrations, an enzymatic hydrolysis step with β-glucuronide should be added before the DLLME procedure. A comparison of examples of sample preparation techniques and chromatographic methods used in the biomonitoring of bisphenols in human amniotic fluid is presented in Table 6. 

Our procedure had a low sample volume compared to the examples presented in Table 6, which is vital for biomonitoring low-availability samples. We used HPLC-FLD as an interesting alternative to LC-MS or GC-MS because some bisphenols exhibit strong fluorescent properties, which enables analysis at concentration levels similar to or lower than those used with an MS detector. Moreover, thanks to the specificity of the FL detector (most matrix interferences do not have fluorescent properties), very high sample purity might not be required during analysis.

## 5. Conclusions

In summary, despite a growing number of published papers about the biomonitoring of bisphenols in various biological and environmental samples, research comparing the concentration levels of bisphenols in human amniotic fluid to their levels in other biological matrices, such as urine or blood, is still very scarce.

Analysis of bisphenols in human amniotic fluid samples is essential to estimate exposure to these xenobiotics among the most vulnerable group—fetuses. Constant biomonitoring with the use of new, green, and fast methods, such as those proposed in this paper, will lead to updating of current restrictions, making products for everyday use safer and preventing future negative health effects, both known (as described in the Introduction) and yet undiscovered.

## Figures and Tables

**Table 1:** Structures and physicochemical properties of studied bisphenols.

No.	Bisphenol	IUPAC Name ^1^	Chemical Structure	Log *p* ^1^	pKa ^1^
**1**	BPF	4-[(4-hydroxyphenyl)methyl]phenol	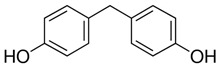	2.9	pKa1 = 7.55 pKa2 = 10.80
**2**	BPE	4-[1-(4-hydroxyphenyl)ethyl]phenol	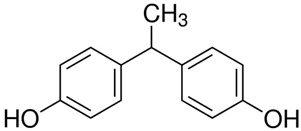	3.9	-
**3**	BPAF	4-[1,1,1,3,3,3-hexafluoro-2-(4-hydroxyphenyl)propan-2-yl]phenol	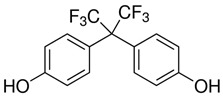	4.5	9.2
**4**	BADGE∙ 2HCl	1-chloro-3-[4-[2-[4-(3-chloro-2-hydroxypropoxy)phenyl]propan-2-yl]phenoxy]propan-2-ol	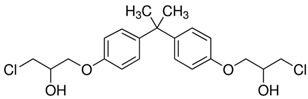	4.6	-
**5**	BPP	4-[2-[4-[2-(4-hydroxyphenyl)propan-2-yl]phenyl]propan-2-yl]phenol	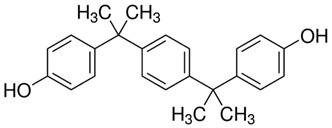	6.1	-

^1^—Data obtained from PubChem database.

**Table 2:** Comparison of variants, recoveries, and relative standard deviations (*n* = 6) obtained during preliminary studies.

Bisphenol	3 mL of Acetone: Dichloromethane (2:1, *v*/*v*)		3 mL of Acetone: Chloroform (2:1, *v*/*v*)		3 mL of Acetone: Dichloromethane: Chloroform (4:1:1, *v*/*v*/*v*)	
	Recovery	RSD%	Recovery	RSD%	Recovery	RSD%
**BPF**	82.6%	11%	87.9%	9%	84.2%	9%
**BPE**	81.7%	8%	94.3%	8%	88.8%	10%
**BPAF**	107.2%	9%	105.5%	11%	97.6%	7%
**BADGE∙2HCl**	101.4%	10%	103.9%	5%	99.7%	16%
**BPP**	100.8%	14%	106.7%	11%	102.3%	13%

**Table 3:** The mean recovery values and RSD% of the studied bisphenols in spiked amniotic fluid samples.

No.	Bisphenol	Recovery Values (%) ± RSD% for Spiking Level (ng/mL)			
		20	30	40	50
**1**	BPF	104.9 ± 9	98.9 ± 9	109.2 ± 9	87.9 ± 9
**2**	BPE	115.9 ± 6	105.1 ± 5	105.1 ± 5	94.3 ± 8
**3**	BPAF	113.3 ± 7	97.0 ± 6	96.2 ± 7	106.7 ± 11
**4**	BADGE∙2HCl	80.9 ± 6	98.9 ± 5	109.2 ± 7	87.9 ± 5
**5**	BPP	99.0 ± 5	105.1 ± 12	105.1 ± 6	94.3 ± 11

**Table 4:** Method validation parameters used for calibration curves in averaged amniotic fluid blank sample: retention times, calibration curve equations, standard deviations of slopes and intercepts, determination coefficients (R^2^), limits of detection (LODs), limits of quantification (LOQs), and confidence levels.

No.	Bp.	Retention Time, t_R_ (min)	Linear Regression	Standard Deviation (SD) of Slope	SD of Intercept	Coefficient of Determination (R^2^)	LOD (ng/mL)	LOQ (ng/mL)	Confidence Level
**1**	BPF	~10.13	y = 5.875x − 5.675	0.31	7.66	0.9916	4.30	13.04	0.95
**2**	BPE	~11.27	y = 6.0876x + 3.7012	0.24	5.95	0.9953	3.23	9.77	0.95
**3**	BPAF	~15.38	y = 14.956x − 12.788	1.38	33.99	0.9816	7.50	22.72	0.95
**4**	BADGE∙2HCl	~18.15	y = 16.592x + 86.834	0.14	3.37	0.9998	2.04	6.17	0.95
**5**	BPP	~19.79	y = 9.2674x + 471.42	0.42	10.38	0.9938	3.70	11.20	0.95

**Table 5:** Comparison of examples of previously published DLLME procedures for biomonitoring of bisphenols with the method described in this work.

Analyzed Bisphenols	Sample Type	Instrumental Analysis	Sample Volume	Extracting Mixture (Dispersive Solvent + Extracting Solvent)	LOD/LOQ	Ref.
**BPA, BPS, BPF, BPZ, BPP, BPAF, BPAP**	Urine	LC-MS/MS	5 mL of urine + 5 of mL water	750 µL of acetone + 500 µL of 1,2-dichloroethane	LOQ: 0.03–0.2 ng/mL	[27]
**BPA, BPF, BPZ, BP**	Urine	GC-MS	2 mL of urine + 8 of mL water	1 mL of acetone + 100 µL chloroform	LOD: 0.01–0.04 ng/mL	[28]
**BPA, BPS, BPP, BPAP, BPAF, BPZ**	Saliva	LC-MS/MS	500 µL of saliva + 500 µL of water	1.5 mL of acetone + 500 µL of chloroform	LOQ: 0.05–0.4 ng/mL	[29]
**BPF, BPE, BPA, BPB, BPAF, BADGE∙2HCl, BPP**	Amniotic fluid collected during amniocentesis	HPLC-FLD	400 µL of amniotic fluid + 1.6 mL of water	2 mL of acetone + 1 mL of chloroform	LOD: 2.04–7.5 ng/mL	This work

Abbreviations: bisphenol S (BPS), biphenyl (BP), bisphenol AP (BPAP), bisphenol Z (BPZ), liquid chromatography–tandem mass spectrometry (LC-MS/MS), gas chromatography–mass spectrometry (GC-MS).

**Table 6:** Examples of biomonitoring bisphenols in human amniotic fluid samples.

Bisphenol	Sample Amount	Extraction Technique Used during Sample Preparation	Chromatographic Method and Detection Technique	LOD/LOQ	Ref.
**BPA**	0.5 mL	SPE (cartridges packed with 200 mg of silica-based bonded C18 material)	LC-MS	LOD = 0.1 ng/mL LOQ = 0.3 ng/mL	[36]
**BPA**	1.0 mL	SPE (C18-based sorbent)	GC-MS	LOD = 0.52 ng/mL	[37]
**BPA, BPP, BPS, BPAF, BPAP**	2.0 mL	Solvent extraction (ethyl acetate)	LC-MS	LOQ: from 0.01 ng/mL to 0.2 ng/mL	[38]
**BPF, BPE, BPAF, BADGE∙2HCl, BPP**	0.4 mL	DLLME	HPLC-FLD	LOQ: from 6.17 to 22.72 ng/mL	This work
